# Hematologic side effects of immune checkpoint inhibitor with or without chemotherapy in patients with advanced and metastatic gastrointestinal cancer: A systematic review and network meta-analysis of phase 3 trials

**DOI:** 10.3389/fphar.2023.1163971

**Published:** 2023-03-22

**Authors:** Jingyi Hou, Ruiyang Xie, Zhuo Zhang, Qianxin Liu, Qian Xiang, Yimin Cui

**Affiliations:** ^1^ Department of Pharmacy, Peking University First Hospital, Beijing, China; ^2^ School of Pharmaceutical Sciences, Peking University Health Science Center, Beijing, China; ^3^ Department of Urology, National Cancer Center/National Clinical Research Center for Cancer/Cancer Hospital, Chinese Academy of Medical Sciences and Peking Union Medical College, Beijing, China; ^4^ Institute of Clinical Pharmacology, Peking University First Hospital, Beijing, China

**Keywords:** hematologic toxicity, immune checkpoint inhibitor, gastrointestinal cancer, phase 3 clinical trial, network meta-analysis

## Abstract

**Background:** The regimens of immune checkpoint inhibitors (ICIs) alone or with chemotherapy are emerging as systemic therapy for patients with advanced and metastatic gastrointestinal cancers. However, the risk of treatment-related hematologic toxicity stays unclear.

**Methods:** We enrolled in phase 3 randomized clinical trials (RCTs) comparing PD-1, PD-L1, and CTLA-4 inhibitors in advanced and metastatic gastrointestinal cancers. The incidences of overall treatment-related adverse events (TRAEs), discontinuation, leukopenia, neutropenia, thrombocytopenia, and anemia were extracted for the Bayesian network meta-analysis. Analyses with poor convergence or low incidence were reported as incidences with 95% CIs instead.

**Results:** Sixteen phase 3 RCTs with 9732 patients who received systemic therapy were included. A total of 150 (1.54% [95% CI 1.31–1.80]) treatment-related death events were recorded, whereas 13 (0.13% [95% CI 0.08–0.22]) of them were hematologic. 0.24% (95% CI 0.12–0.48) patients received ICI plus chemotherapy were recorded for hematological deaths, 0.09% (95% CI 0.01–0.23) were for chemotherapy alone, and 0.05% were for ICI alone (95% CI 0.01–0.29). Febrile neutropenia was the most frequent cause of death in ICI with chemotherapy. For grade ≥3 TRAEs, we found nivolumab plus chemotherapy (OR 1.63 [95% CI 0.84–3.17]) had a higher risk than other treatments. Overall, ICI monotherapy led to fewer AEs than chemotherapy-based regimens in the analyses of leukopenia, neutropenia, thrombocytopenia, and anemia. Among the 11 treatments, toripalimab plus chemotherapy possessed the highest risk in any-grade leukopenia (OR 1.84 [95% CI 0.48, 6.82]) and neutropenia (OR 1.71 [95% CI 0.17, 17.40]) respectively. For grade ≥3 hematologic AEs, neutropenia (20.08% [95% CI 18.67–21.56]) related to ICI plus chemotherapy was the most dominant. ICI plus chemotherapy was likely to increase the incidence than dosing these drugs alone.

**Conclusion:** Using ICI alone had a low incidence of causing hematologic mortality and AEs, while the combination with chemotherapy might magnify the side effects. Comprehensively, pembrolizumab plus chemotherapy and sintilimab plus chemotherapy were the safest regimens in terms of leukopenia and neutropenia respectively. This study will guide clinical practice for ICI-based chemotherapy.

**Systematic Review Registration:** PROSPERO, identifier CRD42022380150

## 1 Introduction

Immune checkpoint inhibitors (ICIs) have emerged as an effective therapy for patients with advanced and metastatic gastrointestinal malignancies. Although the phase 3 KEYNOTE-062 study revealed no clinically meaningful benefit in first-line pembrolizumab plus chemotherapy vs. chemotherapy (Shitara et al., 2020), the use of ICIs still improves the survival outcomes in certain circumstances. For patients with higher programmed cell death ligand 1 (PD-L1) combined positive score (CPS) in esophageal and gastric cancer, the combination of ICI and chemotherapy was recommended as a higher category in National Comprehensive Cancer Network (NCCN) guidelines (Janjigian et al., 2021; Sun et al., 2021). Since KEYNOTE-177, pembrolizumab significantly longer progression-free survival (PFS) than chemotherapy as first-line therapy for microsatellite instability-high/mismatch repair-deficient (MSI-H/dMMR) metastatic colorectal cancer (Andre et al., 2020). The phase 2 CheckMate 142 study further demonstrated nivolumab plus low-dose ipilimumab had clinical benefit for MSI-H/dMMR colorectal cancer as well (Lenz et al., 2022). The preferred first-line treatment regimens are based on fluoropyrimidine and platinum which can induce severe hematologic side effects. The RAINFALL study reported the most common grade 3–4 adverse events (AEs) as neutropenia (27%) and anemia (14%) in patients who received fluoropyrimidine and cisplatin (Fuchs et al., 2019). Consistent with the results, Arai *et al* investigated the safety of fluoropyrimidine with platinum in advanced gastric cancer and found high rates in grade 3–4 leukocytopenia (17%), neutropenia (36%), and anemia (19%) as well (Arai et al., 2019). Meanwhile, blockade of programmed cell death 1 (PD-1) and PD-L1 could activate auto-reactive T-cells and auto-antibodies, then lead to a series of immune reactions (Matsumoto et al., 2020). Hematologic immune-related adverse events (irAEs) were less frequent but could be lethal (Tabchi et al., 2016). By constructing a large cohort of patients treated with ICI, previous research revealed the estimated incidence of hematologic irAE was 0.65% (Kramer et al., 2021). However, the risk of hematologic AEs from the combination of ICI and chemotherapy stays unclear.

Fast recognition and management of treatment-related adverse events (TRAEs) are crucial for patients with advanced cancer. Hematologic side effects are common in chemotherapy, thus understanding the potential risk of combining with ICI is necessary. To date, experienced oncologists have built an instructive framework of the solution to hematologic AEs related to chemotherapy (Crawford et al., 2004; Al-Samkari and Soff, 2021). As hematological side effects of ICIs are rare and difficult to diagnose, hematologic toxicities associated with ICI are poorly described (Schneider et al., 2021). Here, we enrolled the published phase 3 randomized clinical trials (RCTs) and conducted a Bayesian network meta-analysis. By analyzing neutropenia, leukopenia, anemia, thrombocytopenia, general AEs, and TRAEs (all-grade, grade ≥3), we provide a safety assessment of hematologic safety of PD-1, PD-L1, and cytotoxic T lymphocyte-associated antigen-4 (CTLA-4) inhibitors in monotherapy and combination with chemotherapy.

## 2 Methods

### 2.1 Literature search strategy and study selection

PubMed, Cochrane, and Embase databases were searched for relevant works. Several key search terms are listed as follows: ‘immune checkpoint inhibitor’, ‘PD-1’, ‘PD-L1’, ‘chemotherapy’, ‘phase 3’, and ‘gastrointestinal cancer’. Papers published before 2 September 2022 were searched and screened for further analysis. The full search criteria are presented in Supplementary Table S2. We conducted this systematic review and network meta-analysis by following the Preferred Reporting Items for Systematic Reviews and Meta-Analyses (PRISMA) statement (Supplementary Table S1). The protocol was registered in the International Prospective Register of Systematic Reviews (PROSPERO CRD42022380150). Study screening was completed by two independent reviewers (JH and RX), and a third reviewer (ZZ) was consulted for any disagreement. The Inclusion criteria for trial selection were as below (1): Phase 3 RCTs enrolled advanced and/or metastatic gastric cancer, esophageal cancer, gastroesophageal junction cancer, and colorectal cancer (2); The intervention arms must include ICI (PD-1, PD-L1, or CTLA-4 inhibitor) and chemotherapy (3); Detailed data on hematologic and overall AEs were reported (4); Studies were published in English. Studies not meeting the inclusion criteria were excluded. Other exclusion criteria were as below (1): Trials involved treatments other than ICI and chemotherapy (2); Studies exploring the efficacy and safety of sequential treatments (3); Literature such as case reports, cohort studies, conference abstracts, and letters were all excluded.

### 2.2 Data extraction and quality assessments

The following information was collected from each included study: study name, National Clinical Trial number, start year, study objective, treatment line, sample size, intervention regimens, overall TRAEs, treatment-related discontinuation, hematologic AEs (leukopenia, neutropenia, thrombocytopenia, and anemia), and death associated with hematologic AEs. AEs in any-grade and grade≥3 were defined as grade 1–5 and grade 3–5 respectively. TRAEs are defined as any AEs that confirmed by the investigators and might be caused by the study medication with reasonable possibility. All AEs are in accordance with the Common Terminology Criteria for Adverse Events (CTCAE) version 5.0 (Freites-Martinez et al., 2021). We used the Cochrane Risk of Bias Tool to determine risk of the bias in each trial as high, unclear, or low (Higgins et al., 2011). Several score categories were noted: random sequence generation, allocation concealment, the blinding of participants and personnel, incomplete outcome data, the blinding of outcome assessment, selective reporting, and other biases (Supplementary Figure S1). Two authors (JH and RX) independently completed the process, and any disagreements in the assessment were resolved by a third investigator (QL).

### 2.3 Statistical analysis

To determine the appropriate model for network meta-analyses, we used a conservative approach to deal with between-study heterogeneity. If significant heterogeneity existed, we used the fixed effects model; otherwise, we used the Bayesian random-effects consistency model (Mills et al., 2013). Bayesian network modeling gives advantages to adapting to complex situations, by providing a straightforward method for probabilistic statements and treatment effect prediction (Salanti et al., 2011). The incidence of AEs was reported as an incidence with 95% confidence intervals (CIs), estimated through binomial probability. Odds ratios (ORs) with 95% CIs were used to analyze rate outcomes for data of AEs and discontinuation events. The inconsistency of evidence was shown in the inconsistency model comparisons (Lu and Ades, 2006). The surface under cumulative ranking curve (SUCRA) analysis was performed to calculate the AE ranking probability of each treatment regimen (Salanti et al., 2011). Between-study heterogeneity was estimated by the I^2^ values of the consistency model if more than one comparison existed. Ι^2^ values higher than 25%, 50%, or 75% suggested low, moderate, or high heterogeneity, respectively (Higgins et al., 2003).

To visualize the sample size and the number of comparisons, we used the “rjags” and “GeMtc” packages in R 4.0.3 (https://www.r-project.org/) and generated the Bayesian network modeling of AEs (Neupane et al., 2014). Incidences with 95% CI was calculated with the binconf () function in the “Hmisc” package. We also ran the analyses of heterogeneity and ranking probability in R. To identify the heterogeneity effects, the number of adaptations was set to 5000, and the sample iteration parameter was adjusted to 20,000.

## 3 Results

### 3.1 Eligible studies and baseline characteristics

**FIGURE 1:**
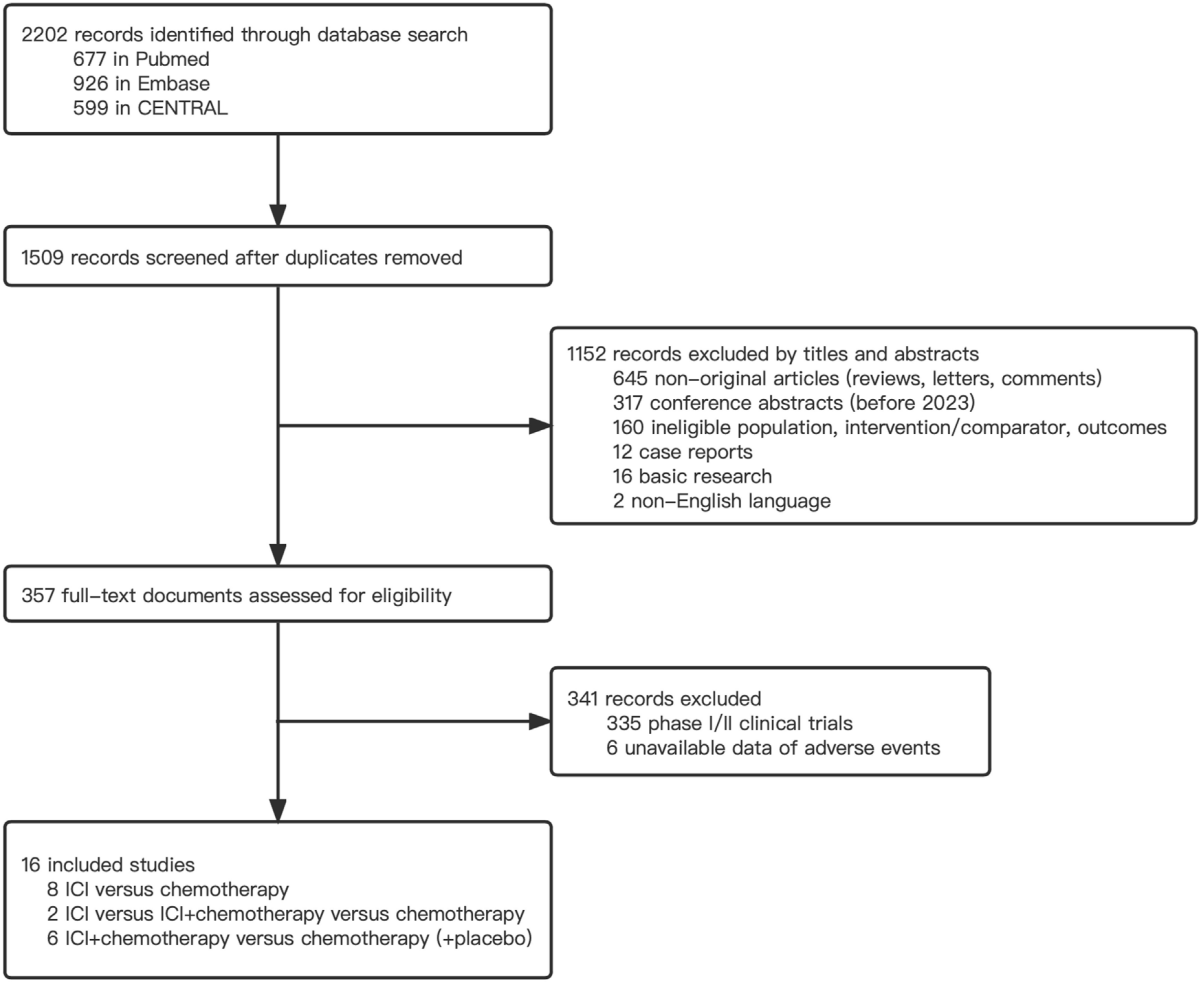
Flow chart of the study selection.

The comprehensive search strategy identified 2202 records, and 357 records were eligible for further full-text screening (Figure 1). Following the selection criteria, 16 phase 3 RCTs with 9732 patients were included in the network meta-analysis (Bang et al., 2018; Shitara et al., 2018; Kato et al., 2019; Andre et al., 2020; Huang et al., 2020; Kojima et al., 2020; Shitara et al., 2020; Janjigian et al., 2021; Luo et al., 2021; Moehler et al., 2021; Sun et al., 2021; Chung et al., 2022; Doki et al., 2022; Kang et al., 2022; Lu et al., 2022; Wang et al., 2022). Among them, 3275 patients received ICI plus chemotherapy, 1926 patients received ICI alone, and 4531 received chemotherapy alone. Nine trials reported first-line therapy, six reported second-line therapy, and one reported third-line therapy. Most studies (15 of 16) investigated advanced and metastatic upper gastrointestinal tract cancer (esophageal cancer, gastroesophageal cancer, and gastric cancer), whereas only one study was associated with lower colorectal cancer. We identified ICIs as PD-1 inhibitors (pembrolizumab, nivolumab, camrelizumab, toripalimab, and sintilimab), PD-L1 inhibitors (avelumab), and CTLA-4 inhibitor (ipilimumab). The main characteristics of the included studies are presented in Table 1.

**TABLE 1:** Studies evaluating safety of immune checkpoint inhibitors with or without chemotherapy.

	Study	Start year	Treatment line	Study objective	Treatment regimen (arm1/arm2/arm3)	No. of patients in safety assessment	Median age	ECOG PS 1
1	KEYNOTE-061 (NCT02370498)	2015	First-line	Advanced gastric or gastroesophageal junction cancer	Pembrolizumab/chemotherapy	294/276	63/60	169/158
2	KEYNOTE-062 (NCT02494583)	2015	Second-line	Advanced gastric cancer	Pembrolizumab/pembrolizumab plus chemotherapy/chemotherapy	254/250/244	61/62/63	125/138/135
3	KEYNOTE-063 (NCT03019588)	2017	Second-line	Advanced gastric or gastroesophageal junction cancer	Pembrolizumab/chemotherapy	47/44	61/61	33/35
4	KEYNOTE-177 (NCT02563002)	2016	Second-line	Advanced colorectal cancer	Pembrolizumab/chemotherapy	153/143	63/63	78/70
5	KEYNOTE-181 (NCT02559687)	2015	First-line	Advanced esophageal cancer	Pembrolizumab/chemotherapy	314/296	63/62	187/197
6	KEYNOTE-590 (NCT03189719)	2017	Second-line	Advanced esophageal cancer	Pembrolizumab plus chemotherapy/placebo plus chemotherapy	370/370	64/62	223/225
7	ATTRACTION-3 (NCT02569242)	2016	First-line	Advanced esophageal squamous cell carcinoma	Nivolumab/chemotherapy	209/208	64/67	109/102
8	ATTRACTION-4 (NCT02746796)	2017	First-line	Advanced or recurrent gastric or gastroesophageal junction cancer	Nivolumab plus chemotherapy/placebo plus chemotherapy	359/358	64/65	167/168
9	CHECKMATE 648 (NCT03143153)	2017	First-line	Advanced esophageal squamous-cell carcinoma	Nivolumab plus chemotherapy/nivolumab plus ipilimumab/chemotherapy	310/322/304	64/63/64	171/174/170
10	CHECKMATE 649 (NCT02872116)	2017	First-line	Advanced gastric, gastroesophageal junction, and esophageal adenocarcinoma	Nivolumab plus chemotherapy/chemotherapy	782/767	62/61	462/452
11	ESCORT-1st (NCT03691090)	2018	Second-line	Advanced or metastatic esophageal squamous cell carcinoma	Camrelizumab plus chemotherapy/placebo plus chemotherapy	298/297	62/62	227/232
12	ESCORT (NCT03099382)	2017	First-line	Advanced or metastatic esophageal squamous cell carcinoma	Camrelizumab/chemotherapy	228/220	60/60	182/176
13	JAVELIN Gastric 100 (NCT02625610)	2015	Third-line	Advanced or metastatic gastric or gastroesophageal junction cancer	Avelumab/chemotherapy	243/238	62/61	147/142
14	JAVELIN Gastric 300 (NCT02625623)	2015	First-line	Advanced gastric or gastroesophageal junction cancer	Avelumab/chemotherapy	184/177	59/61	119/124
15	JUPITER-06 (NCT03829969)	2019	First-line	Advanced esophageal squamous cell carcinoma	Toripalimab plus paclitaxel and cisplatin/placebo plus chemotherapy	257/257	63/62	191/189
16	ORIENT-15 (NCT03748134)	2018	Second-line	Advanced or metastatic esophageal squamous cell carcinoma	Sintilimab plus chemotherapy/placebo plus chemotherapy	327/332	63/63	250/251

Abbreviations: ECOG, eastern cooperative oncology group; PS, performance status.

### 3.2 Overall incidence and cause of treatment-related deaths

To fully describe the landscape of treatment-related death events, we calculated the incidences of overall deaths and hematologic deaths. As shown in Table 2, a total of 150 (1.54% [95% CI 1.31–1.80]) treatment-related death events were recorded, whereas 13 (0.13% [95% CI 0.08–0.22]) of them were hematologic. Febrile neutropenia (0.06% [95% CI 0.03–0.13]) was the most frequent cause of death in ICI-based chemotherapy arms. By setting the population as a patient group who received allocated treatment, eight were correlated with ICI plus chemotherapy (0.24% [95% CI 0.12–0.48]), four were with to chemotherapy alone (0.09% [95% CI 0.01–0.23]), and only one was related to ICI alone (0.05% [95% CI 0.01–0.29]). The incidences of other hematologic TRAEs, including the decrease of white blood cells (WBCs), neutrophils, hemoglobin, and platelet were 0.01% (95% CI 0.01–0.06).

**TABLE 2:** Cause summary of death due to hematologic adverse events.

Drugs	Cause of TRAE death	Number	Study
Pembrolizumab	Decreased WBC count	1	KEYNOTE-181
Pembrolizumab plus chemotherapy	Febrile neutropenia	2	KEYNOTE-062
			KEYNOTE-590
Nivolumab plus chemotherapy	Febrile neutropenia	3	ATTRACTION-4
			CHECKMATE 649
Camrelizumab plus chemotherapy	Anemia	1	ESCORT-1st
Sintilimab plus chemotherapy	Myelosuppression	1	ORIENT-15
	Decrease in platelet count	1	
Chemotherapy with or without placebo	Decreased neutrophil count	1	KEYNOTE-181
	Febrile neutropenia	1	KEYNOTE-590
	Hemolytic anemia	1	ATTRACTION-4
	Decreased platelet count	1	ORIENT-15
Total		13	

Abbreviations: TRAE, treatment related adverse event; WBC, white blood cell.

### 3.3 Network meta-analysis with the consistency and inconsistency model

**FIGURE 2:**
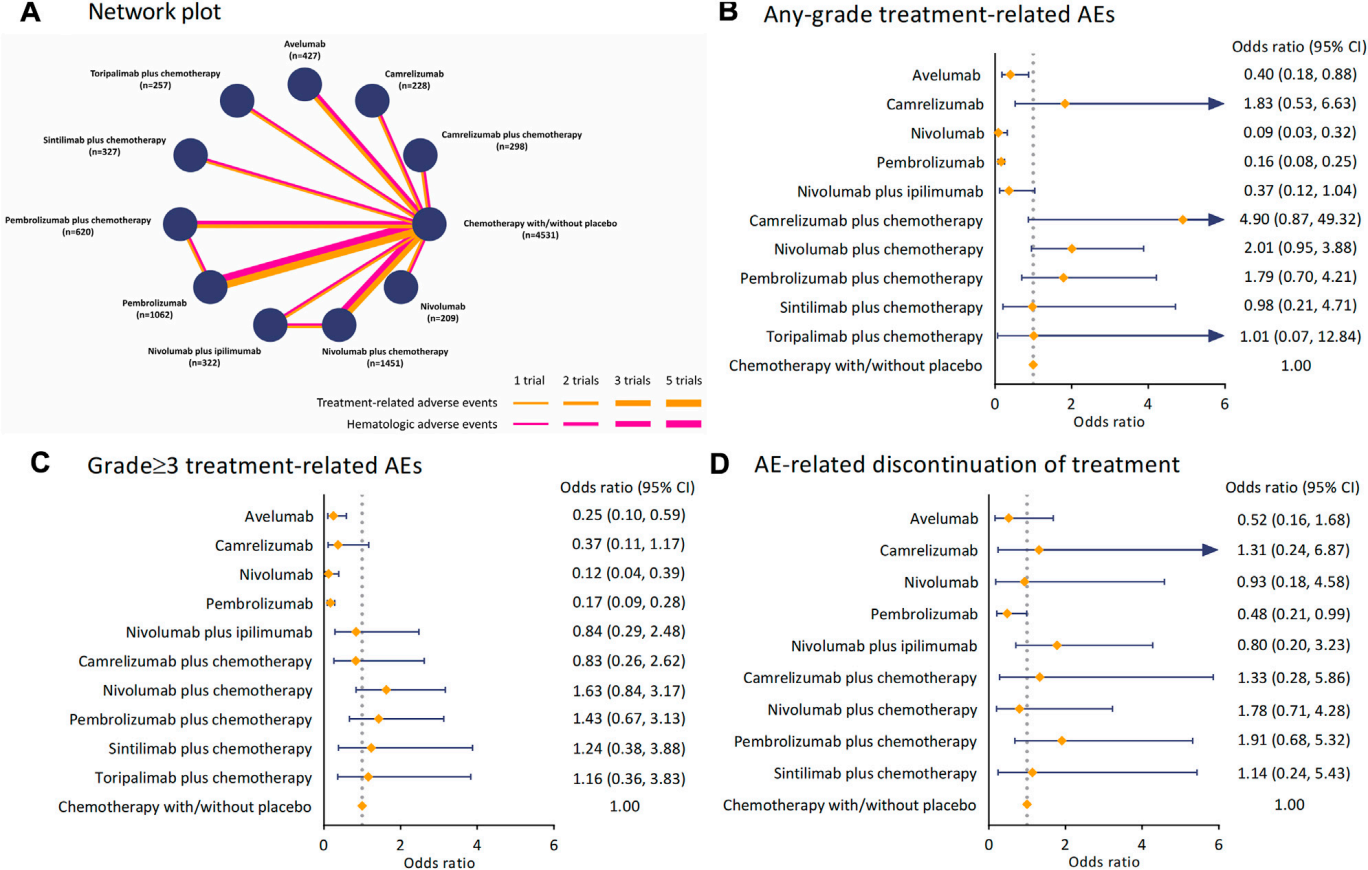
Network plot and pooled estimates of comparisons of TRAEs and AE-related discontinuation of treatment from ICIs. **(A)** Network plots for 16 studies with hematological safety assessment in 11 interventions. Each node refers to a treatment, and each line represents a type of head-to-head comparison. **(B)** ORs with 95% CIs for any-grade TRAEs. **(C)** ORs with 95%CIs for grade≥3 TRAEs. **(D)** ORs with 95%CIs for AE-related discontinuation of treatment. Abbreviation: TRAE, treatment-related adverse event; ICI, immune checkpoint inhibitor; OR, odds ratio; CI, confidence interval; AE, adverse event.

Figure 2A illustrates the general network plots for 16 studies with hematologic safety assessment in 11 treatment regimens. The arms of chemotherapy alone and placebo plus chemotherapy were stratified into a control arm for not receiving any ICI-based treatment interventions. As shown in Figure 2B, the OR of camrelizumab plus chemotherapy *versus* the control arm was 4.90 (95% CI 0.87–49.32) for TRAEs of any-grade, whereas the risk of sintilimab plus chemotherapy (OR 0.98 [95% CI 0.21–4.71]) and toripalimab plus chemotherapy (OR 1.01 [95% CI 0.07–12.84]) were consistent with chemotherapy. In terms of grade≥3 TRAEs, compared with the control arm, the combination of nivolumab (OR 1.63 [95% CI 0.84–3.17]) or pembrolizumab (OR 1.43 [95% CI 0.67–3.13]) with chemotherapy had an increased risk (Figure 2C). Using avelumab (OR 0.25 [95% CI 0.10–0.59]), camrelizumab (OR 0.37 [95% CI 0.11–1.17]), nivolumab (OR 0.12 [95% CI 0.04–0.39]), or pembrolizumab (OR 0.17 [95% CI 0.09–0.28]) alone deemed lower risk than chemotherapy. For AE-related discontinuation of treatment, pembrolizumab plus chemotherapy had the highest OR *versus* the control arm among the regimens (OR 1.91 [95% CI 0.68–5.32]), while sintilimab plus chemotherapy seemed to be the safest (OR 1.14 [95% CI 0.24–5.43]). The analyses of TRAEs of any grade, grade≥3 TRAEs, and discontinuation for AE were performed in inconsistency model to overcome the effect of heterogeneity.

**FIGURE 3:**
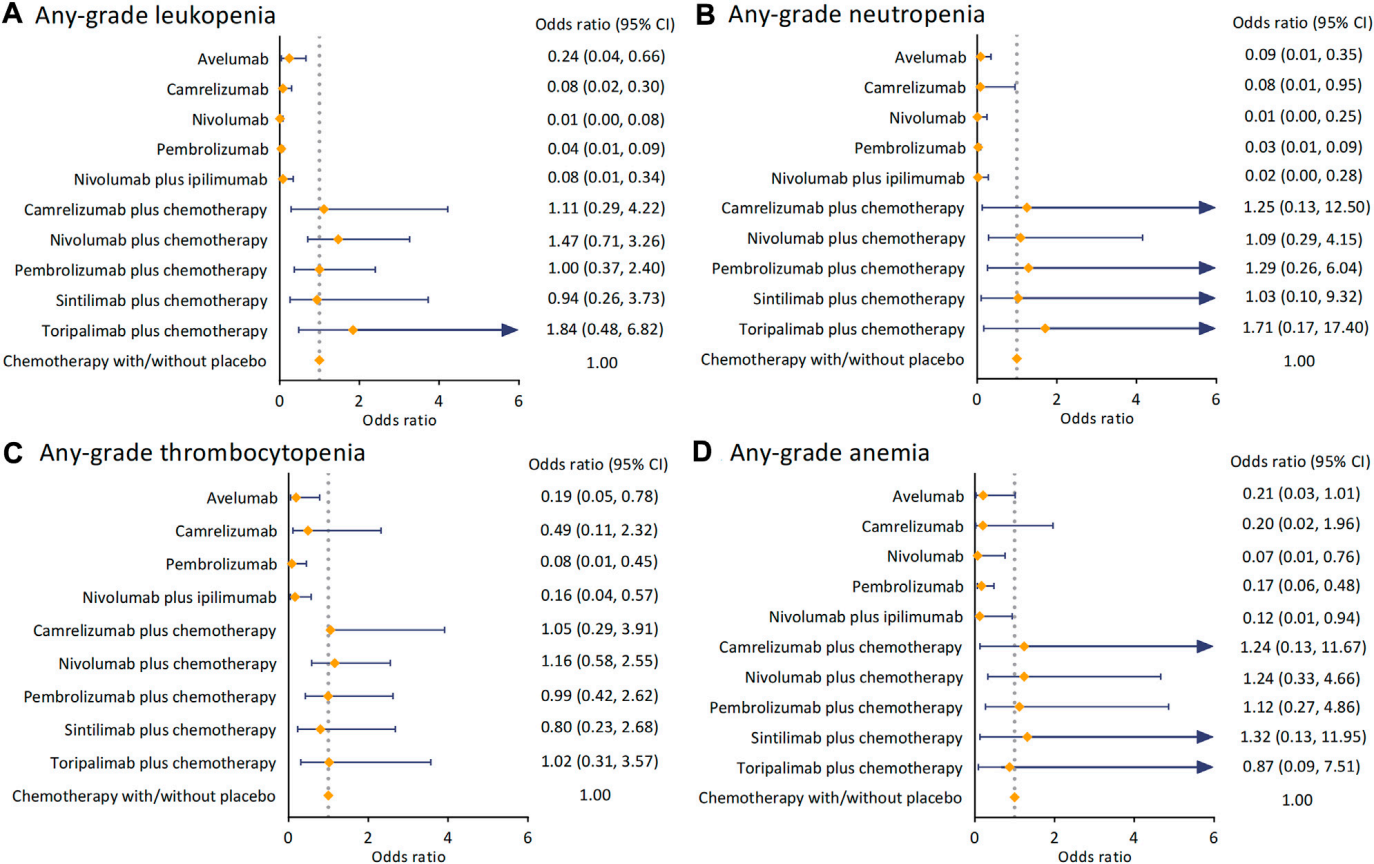
Pooled analysis of any-grade hematological AEs. **(A)** ORs with 95%CIs for any-grade leukopenia. **(B)** ORs with 95%CIs for any-grade neutropenia. **(C)** ORs with 95% CIs for any-grade thrombocytopenia. **(D)** ORs with 95% CIs for any-grade anemia. Abbreviation: AE, adverse event; OR, odds ratio; CI, confidence interval.

We investigated the hematologic side effects by analyzing leukopenia, neutropenia, thrombocytopenia, and anemia of any grade (Figure 3). Overall, compared to chemotherapy, giving ICI alone or in dual had a significantly lower risk of arising hematologic AEs in these four terms. For leukopenia, toripalimab plus chemotherapy increased the risk most (OR 1.84 [95% CI 0.48–6.82]), while pembrolizumab (OR 1.00 [95% CI 0.37–2.40]) and sintilimab (OR 0.94 [95% CI 0.26–3.73]) plus chemotherapy harbored similar ORs when comparing to the control arm. The combination of toripalimab and chemotherapy also caused more neutropenia events (OR 1.71 [95% CI 0.17–17.40]). In contrast, the ORs of nivolumab (OR 1.09 [95% CI 0.29–4.15]) and sintilimab (OR 1.03 [95% CI 0.10–9.32]) plus chemotherapy *versus* the control arm were significantly lower. In terms of thrombocytopenia, all regimens of ICI and chemotherapy were deemed not significantly increased the risk of hematologic side effects. The ORs *versus* chemotherapy ranged from 0.80 (95% CI 0.23–2.68) in sintilimab regimen to 1.16 (95% CI 0.58–2.55) in nivolumab regimen. We found camrelizumab with chemotherapy (OR 1.24 [95% CI 0.13–11.67]) and nivolumab with chemotherapy (OR 1.24 [95% CI 0.33–4.66]) had a consistent risk of causing anemia. Sintilimab plus chemotherapy increased this risk to OR 1.32 (95% CI 0.13–11.95).

### 3.4 Incidences of safety events in ICI with or without chemotherapy

**FIGURE 4:**
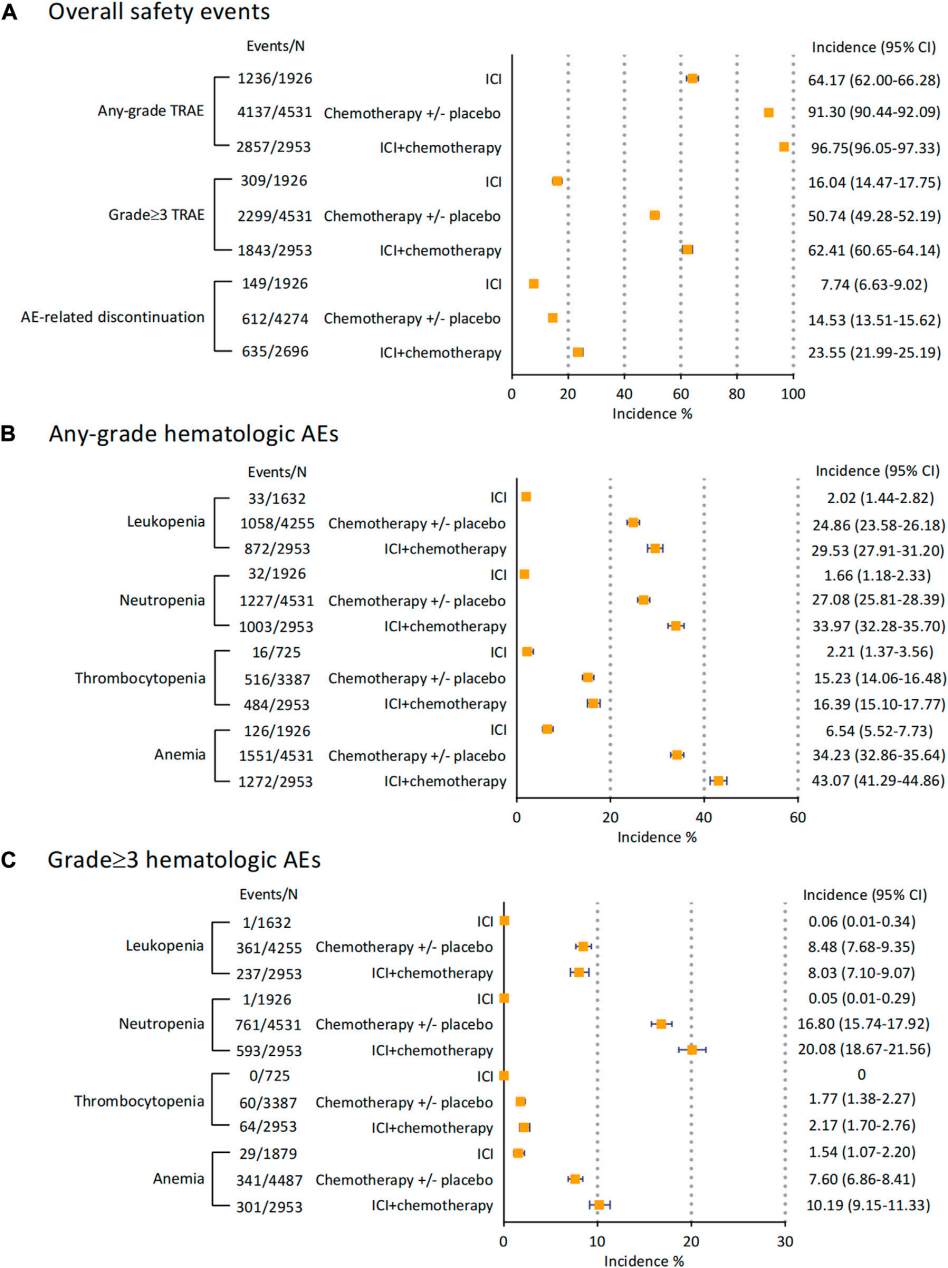
Incidences of overall safety events and hematological AEs of ICI monotherapy, ICI combined with chemotherapy, and chemotherapy with/without placebo. **(A)** incidences of overall safety events. **(B)** incidences of any-grade hematological AEs. **(C)** incidences of grade 3 hematological AEs. Abbreviation: AE, adverse event; ICI, immune checkpoint inhibitor.

To explore the potential additive safety risk of combination therapy, we stratified all the treatments (ICI, Chemotherapy +/- placebo, and ICI plus chemotherapy). The incidences of overall safety events and any-grade hematologic AEs were separately recorded and seen in Figure 4. The combination of ICI and chemotherapy caused more grade ≥3 TRAEs than using ICI or chemotherapy alone (Incidence 62.41% [95% CI 60.65–64.14]), as well as AE-related discontinuation events (Incidence 23.55% [95% CI 21.99–25.19]). By examining the emergence of leukopenia, neutropenia, thrombocytopenia, and anemia (Figure 4B), we found ICIs were associated with 2.02% (95% CI 1.44–2.82) - 6.54% (95% CI 5.52–7.73) any-grade AEs only. Giving chemotherapy caused 24.86% (95% CI 23.58–26.18) of patients with leukopenia and 27.08% (95% CI 25.81–28.39) with neutropenia, respectively. These incidences raised to 29.53% (27.91–31.20) for leukopenia and 33.97% (32.28–35.70) for neutropenia when dosing ICI with chemotherapy. Notably, giving ICI alone barely caused any grade≥3 leukopenia, neutropenia, and thrombocytopenia (Figure 4C). The most frequent grade≥3 hematologic AE for chemotherapy alone was neutropenia (Incidence 16.80% [95% CI 15.74–17.92]), and the combination therapy increased the incidence to 20.08% (95% CI 18.67–21.56). For grade ≥3 anemia, ICI with chemotherapy was accounted for 10.19% (95% CI 9.15–11.33) AEs. However, the incidences of grade≥3 leukopenia (Incidence 8.03% [95% CI 7.10–9.07]) and thrombocytopenia (Incidence 2.17% [95% CI 1.70–2.76]) in regimens of ICI and chemotherapy were almost consistent with chemotherapy alone.

### 3.5 Assessment of inconsistency, heterogeneity, and risk of bias

We calculated the variance deviation of random effects and inconsistency model and then presented the results in Supplementary Table S3. The heterogeneity of general AEs and hematologic AEs was estimated and shown in Supplementary Table S4. The Ι^2^ value suggested high heterogeneity in the analysis of anemia (Ι^2^ = 83.6) and moderate heterogeneity in TRAEs of any grade (Ι^2^ = 50.3), grade≥3 TRAEs (Ι^2^ = 72.3), and discontinuation for AE (Ι^2^ = 63.1). We assessed the quality assessment scored by the Cochrane risk of bias tool in Supplementary Figure S1. Among the 16 studies, 11 of them had high risk of performance bias for poor blinding of participants.

## 4 Discussion

In this systematic review and network meta-analysis of ICI with or without chemotherapy, 16 trials for patients with advanced and metastatic gastrointestinal malignancies were evaluated. We assessed the categorized safety profile of PD-1, PD-L1, CTLA-4 inhibitors, and chemotherapy in ten ICI-based regimens. The general results indicate the principal findings:

(1) ICI caused much fewer general TRAEs and hematological TRAEs than ICI with chemotherapy or chemotherapy alone;
(2) The incidences of treatment-related hematological death were 0.24% in patients who received ICI with chemotherapy and 0.05% in patients received ICI alone;
(3) Febrile neutropenia was the most common cause of death in pembrolizumab plus chemotherapy and nivolumab plus chemotherapy;
(4) Toripalimab plus chemotherapy had the highest risk of leukopenia and neutropenia events, whereas sintilimab plus chemotherapy had the best safety in these two analyses;
(5) The incidence of hematologic AEs in ICI plus chemotherapy was higher than with the simple addition of ICI and chemotherapy.

Hematologic toxicities, commonly observed with chemotherapy, are the results of a cytotoxic effect on hematopoietic stem cells located in the bone marrow. Several cellular elements of the blood, including red blood cells (RBCs), WBCs, and platelets are involved. For decades, chemotherapy has been seen as a crucial regimen in patients with advanced and metastatic gastrointestinal cancer. To maximally ensure efficient dose and controllable tolerability, clinicians have greatly explored the hematologic side effects induced by chemotherapy and summarized a series of strategies (Ferreira et al., 2017; Castaman and Pieri, 2018). As ICIs are often given with chemotherapy (fluorouracil, capecitabine, oxaliplatin, cisplatin, *etc.*) in metastatic gastrointestinal cancer, understanding the potential hematological risk that combination therapy may arise can improve clinical practice.

Treatment-related death events are the most severe outcomes in the clinical experience. Among the 1926 patients who received ICI alone, only one death was recorded in pembrolizumab arm for decreased WBC count. Kramer *et al.* investigated hematological irAEs by enrolling 7626 patients treated with ICI, and only one had fatal outcomes (Kramer et al., 2021). Wang *et al.* explored the safety in 20,128 patients who received PD-1 and PD-L1 inhibitors, and the hematologic death rate was about 0.02% (Wang et al., 2019). Even though the hematological mortality of ICI is rare, clinicians should be aware of the potential side effects of the increased use of ICI. An observational study indicated both the low frequency of hematological toxicities (less than 1% in patients treated with anti-PD(L)-1) and the high rate of serious cases (grade ≥4 in 77% of patients) (Delanoy et al., 2019). Hematologic toxicities caused by ICI are divided into immune and non-immune. To date, no efficient technique has been reported to distinguish whether the hematological AEs are immune-related, which is crucial to the following treatment. Hematologic irAEs are highly life-threatening adverse reactions with a mortality rate reported to be 14% (Michot et al., 2019). The lethal causes of hematologic irAEs were identified as pancytopenia or aplastic anemia, autoimmune hemolytic anemia, hemophagocytic syndrome, and pure red cell aplasia.

The combination of ICI and chemotherapy may have additive hematologic side effects than using ICI or chemotherapy alone. Several meta-analyses explored the hematologic safety and tolerability of ICIs and chemotherapy respectively. Using PD-1/PD-L1 inhibitors alone, the rates of high-grade hematologic AEs were 0.2% for neutropenia, 0.5% for anemia, and 0.2% for thrombocytopenia (Nishijima et al., 2017). Using chemotherapy alone, the rates of high-grade hematologic AEs were 12.3% for neutropenia, 3.0% for anemia, and 3.4% for thrombocytopenia (Nishijima et al., 2017). Notably, when combined with chemotherapy, the rates of grade 3–5 hematologic AEs were higher than the summation of these two regimens (19.6% for neutropenia, 11.4% for anemia, and 6.8% for thrombocytopenia) (Zhou et al., 2021). Consistently, we found the combination of ICI and chemotherapy had a high incidence of leukopenia (29.53% [95% CI 27.91–31.20]), neutropenia (33.97% [95% CI (32.28–35.70]), and anemia (43.07% [95% CI 41.29–44.86]). Petrelli *et al.* enrolled 9324 patients with pan-cancer who received PD-1 and PD-L1 inhibitors, and indicated that severe neutropenia, thrombocytopenia, and febrile neutropenia were rare (Petrelli et al., 2018). ICIs were correlated with a moderate risk of anemia (10%) and a low risk of neutropenia and thrombocytopenia (0.9% and 2.8%), with negligible risk of febrile neutropenia (0.45%) (Petrelli et al., 2018). In the mortality analysis of this study, the hematological mortality of patients treated with chemotherapy alone was 0.09% (4/4531). However, the incidence of hematological death rose to 0.24% when combining ICI with chemotherapy. Unlike the non-specific cytotoxic effect of chemotherapy, PD-1/PD-L1 blockers have identical inhibitory effects on T-lymphocyte classes, B lymphocytes, NK cells, and macrophages. As a result, the putative mechanisms of ICI-associated hematological toxicities are described as autoantibody production, direct cytotoxicity, and excessive cytokine production (Kroll et al., 2022). The finds suggested that using ICI with chemotherapy needed careful estimation and caution for hematological safety. Here, by comprehensively analyzing, we present the hematological TRAEs that should be concerned when giving the regimes of each ICI plus chemotherapy.

- Pembrolizumab plus chemotherapy: neutropenia
- Nivolumab plus chemotherapy: leukopenia, anemia
- Camrelizumab plus chemotherapy: neutropenia, anemia
- Sintilimab plus chemotherapy: anemia
- Toripalimab plus chemotherapy: leukopenia, neutropenia

To date, no study has provided the hematologic safety profile of ICI with or without chemotherapy in advanced gastrointestinal cancer. Previous meta-analyses enrolled clinical trials of all-phase and focused on general safety (Yang et al., 2020; Guo et al., 2022). Our research included phase 3 trials only to avoid the risk of reporting bias and quality control. By describing the incidence and network meta-analysis, we optimized the data presentation and ensured reporting accuracy. However, this work had several limitations that should be stated. First, we observed moderate to high heterogeneity in the analysis of anemia, TRAEs, and discontinuation for AE. The major contribution of heterogeneity was from the ATTRACTION-3 study. A possible reason for heterogeneity presence was the stratification of different chemotherapy regimens, which was designed to construct an entire and clear network. Second, this meta-analysis was performed at the study level instead of analyzing individual data. Third, to ensure drug tolerability, patients enrolled in these trials were screened before the recruitment. Therefore, in real-world experience, the patients may have more comorbidity than those who enrolled in clinical trials, potentially leading to a higher rate of side effects. Due to a very low incidence of hematologic irAEs and only numerical comparisons, the conclusion that ICI with chemotherapy could bring more mortaliteis may alter in future research. Finally, the results might be affected by the open-label design in 11 of 16 trials enrolled in this study, accounting for ascertainment bias.

## 5 Conclusion

In summary, using ICI alone had a low incidence of hematological AEs and mortality, however, with the combination of chemotherapy, the side effects could be magnified. Lethal febrile neutropenia was the most common cause for pembrolizumab and nivolumab with chemotherapy. Regimens of pembrolizumab plus chemotherapy and sintilimab plus chemotherapy were safe in arising leukopenia and neutropenia, respectively. These findings can optimize future trial designs and guide clinical pharmacology for investigations of ICI combination therapy.

## Data Availability

The original contributions presented in the study are included in the article/Supplementary Material, further inquiries can be directed to the corresponding authors.
